# Growth and Neurodevelopment of HIV-Exposed Uninfected Children: a Conceptual Framework

**DOI:** 10.1007/s11904-019-00459-0

**Published:** 2019-11-15

**Authors:** Catherine J. Wedderburn, Ceri Evans, Shunmay Yeung, Diana M. Gibb, Kirsten A. Donald, Andrew J. Prendergast

**Affiliations:** 1 Department of Clinical Research, London School of Hygiene & Tropical Medicine, London, UK; 2 Department of Paediatrics and Child Health, Red Cross War Memorial Children’s Hospital and Neuroscience Institute, University of Cape Town, Cape Town, South Africa; 3 Blizard Institute, Queen Mary University of London, London, UK; 4 Zvitambo Institute for Maternal and Child Health Research, Harare, Zimbabwe; 5 MRC Clinical Trials Unit, University College London, London, UK

**Keywords:** HIV-exposed uninfected, Child, Growth, Stunting, Early child development

## Abstract

**Purpose of Review:**

The population of HIV-exposed uninfected (HEU) children is expanding rapidly, and over one million HEU infants are born each year globally. Several recent studies have reported that HEU children, particularly in low- and middle-income countries, are at risk of poor outcomes, including impaired growth and neurodevelopment. However, the reasons for poor clinical outcomes amongst HEU children remain unclear.

**Recent Findings:**

We summarise the findings from recent large studies that have characterised growth and neurodevelopment in HEU children, identified risk factors and explored underlying mechanistic pathways. We propose a conceptual framework to explain how exposure to HIV and antiretroviral therapy (ART) may lead to adverse growth and neurodevelopment in uninfected children, and review the available evidence and research gaps.

**Summary:**

We propose that HEU children are affected both indirectly, through the augmentation of universal risk factors underlying poor growth and neurodevelopment, and directly through HIV/ART-specific pathways, which ultimately may converge through a series of common pathogenic mechanisms*.* In the era of universal ART, a better understanding of these pathways is crucial to inform future prevention and intervention strategies.

## Introduction

Globally, approximately 1.4 million HIV-infected pregnant women give birth each year [1, 2]. The increased coverage of antiretroviral therapy (ART) for pregnant and breastfeeding women through prevention of mother-to-child transmission (PMTCT) programs has dramatically reduced perinatal and postnatal HIV transmission. Correspondingly, the global population of HIV-exposed uninfected (HEU) children is increasing and, in 2017, was estimated to have reached 14.8 million [3]. This number will continue to increase with improved PMTCT coverage as over one million HEU children are born every year, the majority of whom are now also exposed to ART [3–5]. Emerging data showing poorer health outcomes of HEU compared to HIV-unexposed children [6] means there is a pressing need to understand and address the mechanisms underlying compromised outcomes.

The Sustainable Development Goals have focused attention on the importance of healthy growth and development, so that children can thrive as well as survive. The in utero period and first 2 years after birth (the first 1000 days) represent a highly sensitive period of development, during which substantial physical growth, including brain maturation, occurs [7]. Early fetal exposures are recognised to have long-term consequences for the child and future adult. Given the number of children exposed to HIV and ART in utero, it is important to understand the effects of these exposures. This review will first explore growth and development in HEU children; second, outline a conceptual framework to highlight the pathways through which HIV exposure may impact growth and development; and third, examine how this may help to inform prevention and intervention strategies.

### Studies of HIV-Exposed Uninfected Children

Over recent decades, there has been a growing number of studies evaluating the outcomes of HEU children. However, interpretation of studies can be challenging for several reasons. First, the effects of HIV exposure may differ between high-income and low-income settings. The epidemiology of HIV infection in high-income settings, where adult infections are often found in populations with more substance use and mental health disorders, differs from the more generalised epidemic in sub-Saharan Africa. In addition, HEU children vary in terms of duration of exposure to both HIV (prenatal/postnatal) and ART and are exposed to different antiretroviral drug classes and combinations. This may mean mechanisms differ across countries. Second, studies are often limited by a lack of comparable HIV-unexposed control groups, particularly due to differences in breastfeeding and socioeconomic status. Third, studies have often been small in size without adequate HIV testing of included children to rule out HIV infection. Fourth, there has been a lack of standardised definitions of outcomes. Despite these limitations, it is possible to draw conclusions on health outcomes from large, well-conducted studies, many of which have clearly demonstrated that HEU children have higher mortality than HIV-unexposed children [6, 8–11], predominantly driven by increased frequency and severity of common childhood infections, particularly respiratory disease [6, 8, 9, 12–15]. Recent meta-analyses estimate twofold higher child mortality amongst HEU compared to HIV-unexposed children in the first 1–2 years after birth, with a similar risk persisting in children between 2 and 5 years of age [10, 11]. Finally, a clear divide in the categorisation of studies comes from the use of ART. Studies from the pre-ART era have the benefit of exploring the effect of HIV exposure without the potential confounding effects of ART. However, they may be less relevant in the modern era where most HEU children are also ART-exposed. Although there are clear benefits from ART in reducing HIV transmission and improving maternal health, there is the potential for ART to have negative effects on the developing fetus, and separating the effects of HIV and ART exposure is challenging.

## Growth and Neurodevelopment of HEU Children

**Fig. 1:**
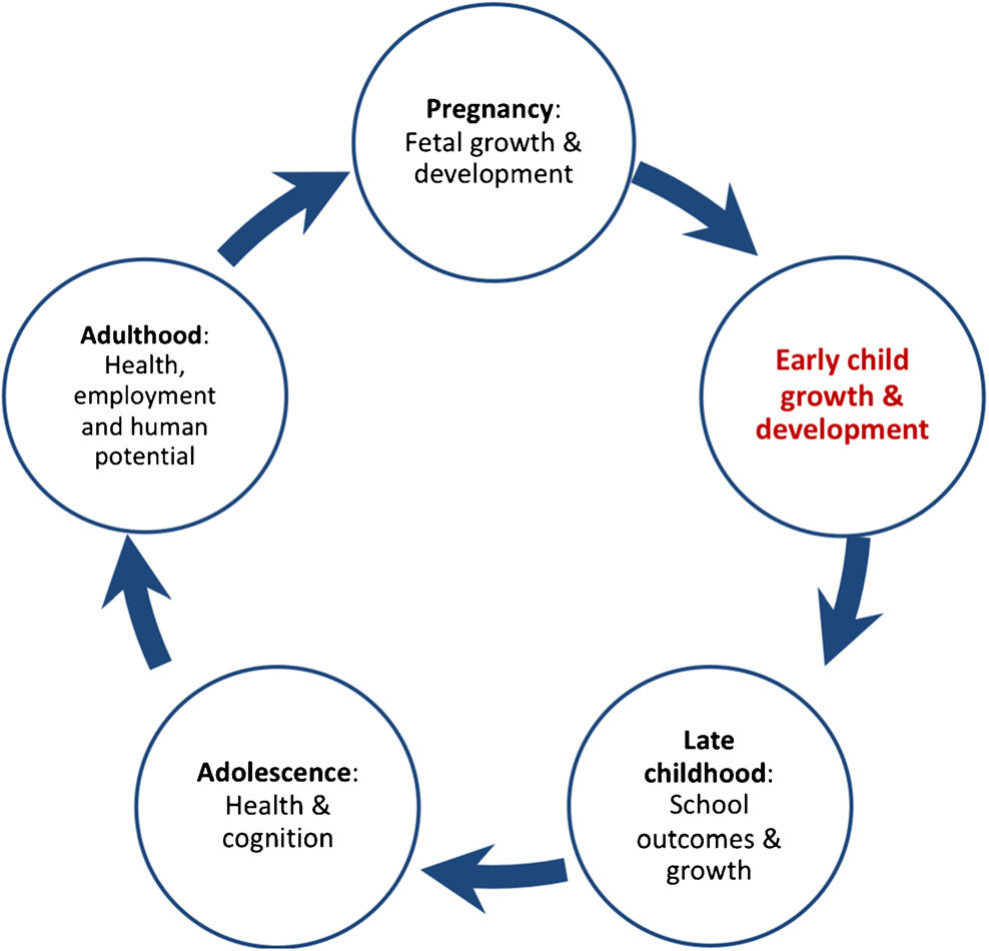
The cycle of child growth and development

An estimated 250 million (43%) children under the age of 5 years fail to reach their developmental potential in low- and middle-income countries (LMIC), based on proxy measures of stunting and poverty [16••]. Impaired growth and development have far-reaching consequences across the life-course, impacting academic outcomes, employment and long-term non-communicable disease risk, as well as intergenerational effects on health and human capital (Fig. 1). Emerging data indicate that this cycle may be critically influenced by HIV exposure.

### Growth Outcomes

Linear growth is an important reflection of overall child wellbeing. In the largest cohort to date, Zimbabwean HEU children in the pre-ART era had 23% more stunting (length-for-age *Z* score < − 2) than HIV-unexposed children from the same communities by 12 months of age [17]. The effect of HIV exposure on growth in the ART era is less clear because there have been few studies to date. Reducing antenatal HIV exposure through ART control of maternal viraemia during pregnancy decreases the risk of transmission and may have additional benefits for HEU infants. However, intrauterine growth restriction and preterm birth have been associated with certain antiretroviral drugs in some studies [18•]. Findings from two large cohorts in the ART era have recently been reported in two different African settings. In Cape Town, where overall stunting prevalence was low, HEU children had lower length-for-age *Z* scores and almost threefold more stunting than HIV-unexposed children [19•]. In rural Zimbabwe, where overall stunting prevalence was high, HEU children also had lower length-for-age *Z* scores and almost twice as much stunting [20]. Similar findings were seen for weight-for-age, underweight (weight-for-age *Z* score < − 2), head circumference and microcephaly (head circumference-for-age *Z*- score < − 2), although not weight-for-length or wasting (weight-for-length *Z* score < − 2) [20]. Collectively, these studies indicate that growth impairment continues to be a major problem amongst HEU children in the current PMTCT era.

### Neurodevelopmental Outcomes

The negative impact of HIV infection on child brain development, both clinically and neuroradiologically, is well-established [21, 22]. There is increasing evidence that HIV exposure (without infection) may also be associated with neurodevelopmental impairment, although the manifestations are more subtle than for HIV-infected children [21, 23, 24••]. Studies from LMIC settings report an impact of HIV exposure on language [21–23, 24••, 25, 26], behaviour [21, 23], cognition [24••, 26] and motor function [24••, 27], although there are limitations to these studies: most were from the pre-ART era, had small sample sizes and lacked adequate comparator groups. A large Zimbabwean study from the pre-ART era found that head circumference was consistently lower in HEU compared to HIV-unexposed infants throughout the first year after birth [28], but there were no neurodevelopmental evaluations in this cohort.

Recent studies in sub-Saharan Africa have generally supported findings from the pre-ART era. One study of South African HEU children aged 12 months found increased odds of cognitive (OR 2.28, 95% CI 1.13, 4.60) and motor delay (OR 2.10, 95% CI 1.03, 4.28) [29•], while another from Botswana showed increased expressive language delay (aOR 1.44, 95% CI 1.01, 2.06) at 2 years [30]. HEU children aged 2 years in rural Zimbabwe had poorer motor and language development compared to their HIV-unexposed community counterparts [31] and the Drakenstein Child Health Study found receptive and expressive language delay in South African HEU children compared to HIV-unexposed children at 2 years of age [32•].

In contrast, a recent study from Uganda and Malawi did not find any neurodevelopmental differences in HEU children aged 1 to 5 years compared to an HIV-unexposed group [33•]. Similarly, evidence from HEU children in high-income settings has generally been reassuring [34, 35]. However, some language delay has been reported [36, 37] and there is a suggestion that cognition and behaviour may be affected at older ages [21, 23] [38]. A UK study found similar outcomes for HIV-infected adolescents and their uninfected siblings, and that both groups were impaired compared to normative data [39]. There are few studies of older HEU children in LMIC settings from the current ART era. One multisite study across five African countries did not find any cognitive differences compared to HIV-unexposed children [40], although language skills were not reported. Other studies have indicated potential ongoing delays, including poorer school mathematics performance in Zambia [41], and lower IQ, language and fine motor development in HEU children aged 2–12 years in Thailand and Cambodia [26].

Overall, although further research is needed, current evidence suggests HEU children may be at risk of delayed neurodevelopment in the early years of life, particularly in LMIC settings, and there are concerns regarding later school and behavioural outcomes.

## Pathogenesis—Universal Pathways Versus HIV-Specific Pathways?

**Fig. 2:**
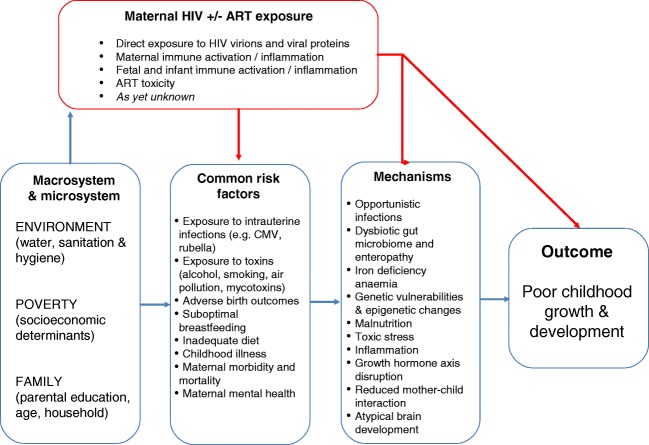
Conceptual framework of the hypothesised pathways through which HIV and ART exposure affect child growth and development. Red lines demarcate HIV-specific pathways; blue lines represent universal pathways. ART, antiretroviral therapy

We hypothesise that there are two broad pathways through which HIV exposure without infection may impact child growth and development: (1) indirectly, by augmenting existing universal pathways that are known common risk factors for poor growth and development; and (2) directly, via HIV-specific mechanisms including exposure to HIV virions, immune activation and ART toxicity **(**Fig. 2**)**.

### Augmentation of Universal Pathways

Growth and development entail the interaction of genetically determined biological processes and environmental influences. The ecological model of development [42] describes the interacting nature of these internal and external factors over time. Both the cumulative burden and timing of these risks likely influence neurodevelopment [43]. Research suggests that HIV exposure may augment a range of universal risk factors, and below, we outline the available evidence.

#### Intrauterine Infections

Many in utero infections have consequences for the developing fetus. Cytomegalovirus (CMV), rubella, Zika virus, syphilis and toxoplasmosis have all been documented to impact child development [44]. HIV-infected mothers are at increased risk for some of these infections; several studies, although not all, have indicated that CMV in particular may be more prevalent in HIV-affected mothers and children [45•, 46] and may explain some of the adverse outcomes associated with HIV exposure [47].

#### Toxins

Exposure to alcohol, tobacco and recreational drugs has a dose-related impact on child growth and development [48, 49]. This is potentially through direct toxicity, augmentation of inflammatory upregulation, as well as poorer maternal health-seeking and parenting behaviour. HIV infection in adults is associated with an accentuated risk of alcohol and substance use in many settings [34]. Air pollution and environmental toxins (such as mycotoxins) are also potential risk factors for poor child growth [50, 51]. There is some evidence that HIV-infected individuals may have higher mycotoxin levels despite the same exposure when compared to HIV-uninfected controls. This may be due to impaired liver function resulting in a decreased ability to detoxify metabolites, though not all reports have corroborated this finding [52, 53]. Mycotoxin exposure may plausibly exacerbate several pathways underlying HIV pathogenesis (including enteropathy and micronutrient deficiencies), thereby affecting early growth [54]. Additionally, the interacting effects of air pollutants and HIV have been associated with adverse birth outcomes, potentially through increasing nitric oxide levels [51].

#### Adverse Birth Outcomes

Prematurity and small-for-gestational age (SGA) are consistently associated with poor postnatal growth and development [55, 56]. Premature infants are vulnerable to prolonged hospitalisation and are at greater risk for neonatal infection and sepsis [57]. HIV-exposed children are at increased risk of prematurity, SGA and low birth weight [58], which are major risk factors for stunting [59]. Evidence suggests that HIV exposure remains associated with adverse birth outcomes and neonatal death despite maternal ART [60, 61]. Separately, ART exposure has been associated with adverse birth outcomes. A recent meta-analysis found that HIV-positive women who conceived on ART had 41% greater risk of preterm delivery compared to HIV-positive women who started ART during pregnancy [18••]. A study from Botswana showed this risk is similar between efavirenz- and dolutegravir-containing regimens [62]; however, recent concerns have been raised around the safety of dolutegravir at the time of conception due to a potential increase in neural tube defects [63]. Further data on the impact of specific drugs and the relationship between poor birth outcomes and timing of ART initiation are urgently needed.

#### Breastfeeding Practices

Global recommendations are that breastfeeding should be early (initiated within 1 h of birth), exclusive (breastmilk and prescribed medications only for the first 6 months of life) and prolonged (through 2 years of age). Suboptimal breastfeeding increases morbidity and mortality, particularly in LMIC settings [64]. A review of 17 observational studies demonstrated that better cognitive outcomes were associated with optimal breastfeeding practices [65]. Findings from a 2015 analysis of the 1982 Pelotas birth cohort in Brazil also reported a dose–response association between breastfeeding duration and increased child cognitive performance, educational attainment and income at the age of 30 years [66]. Globally, guidance on infant feeding for HIV-positive mothers in LMIC settings has changed over time with the introduction of PMTCT and recognition of the benefits of breastfeeding, despite the risk of postnatal HIV transmission [67]. However, formula feeding to prevent postnatal HIV transmission through breastmilk means that HEU children continue to have lower rates of breastfeeding across the world, which may influence developmental outcomes. The impact of postnatal ART exposure through breastfeeding is still to be examined.

#### Inadequate Diet

Food insecurity in pregnancy is a risk factor for adverse birth outcomes and, during early childhood, has been associated with poor growth and neurodevelopment arising from complex mechanisms including nutritional insufficiencies and increased parental stress [68]. Children need sufficient nutrients for growth and immune development, and dietary diversity is necessary for optimal child outcomes. Food insecurity often coexists with HIV infection [69] and has been found to be associated with lower ART adherence, increased HIV-associated illness and decreased survival in HIV-infected individuals [70].

#### Childhood Illness

Illnesses such as childhood pneumonia are associated with long-term physical sequelae [71]. If hospitalisation is needed, this may also have considerable impact on children and their families [72], causing separation from parents and reducing school attendance. Mounting evidence suggests HEU children have increased infectious morbidity [6, 15, 73], and studies to date have found this is particularly due to viral and bacterial respiratory infections [14, 74, 75] in early life. There is also evidence that HEU children are at risk for unusual infections [76], including *Pneumocystis jirovecii* [9, 77–82], CMV [79, 83, 84] and haemorrhagic varicella [12, 77]. Correspondingly, cohort studies from both LMIC and high-income settings suggest HEU children have a higher risk of hospitalisation during infancy [85–87] as well as elevated mortality [10]. In pre-ART era Zimbabwe, morbidity and mortality of HEU children were strongly associated with maternal HIV disease severity, and increased infectious morbidity amongst HEU children remained until maternal CD4 counts were ≥ 800 cells/μL [88]. In the ART era, a recent study from Belgium found that initiation of maternal ART prior to pregnancy (i.e. conception occurred on ART) appeared protective against infectious morbidity in HEU children; however, further work is needed to explore these findings in LMIC [86].

#### Maternal Illness and Death

Maternal physical health before and after birth critically influences child growth and development. Mothers who are unwell may be less able to bond with and care for their children and maternal undernutrition is a risk factor for adverse birth outcomes [59, 89]. Furthermore, the loss of one or more parents, orphanhood and institutionalisation have severe repercussions [90]. There is extensive literature from the pre-ART era on the impact of parental death from HIV/AIDS, and it is estimated over 17 million children have lost one/both parents from the HIV/AIDS epidemic [90]. Parental death may affect children emotionally and impact subsequent relationships. Maternal death is itself related to outcomes of HEU infants [91], potentially for several reasons. First, an infant born to a mother with advanced disease may have greater immune abnormalities; second, mothers who are sick in the late stages of their illness may be unable to care for their children adequately, both physically and emotionally; and third, children who lose their mothers may be subject to extreme poverty and homelessness [92]. Even in the ART era, maternal HIV infection is associated with higher morbidity and mortality, and disease severity in HIV-infected mothers has been associated with infant health outcomes [93].

#### Maternal Mental Health

Maternal psychological illness is linked to adverse child health outcomes. Maternal depression is a risk factor for impaired child growth and development [94, 95]. Similarly, maternal stress has been found to have long-term repercussions on child psychological health [96]. In many communities, there is an association between maternal stress or depression and HIV [97], and the additional exposure to maternal mental health problems has been associated with risk of poor cognitive development in HEU children [98]. Maternal capabilities, which reflect the attributes required to care for a child, may be affected by HIV infection. HIV-infected parents who are unwell may be at risk of poverty because of an inability to work, which may affect availability of food and access to healthcare for the household. HEU children may be expected to take on caregiver roles for infected family members or may be neglected when other children in the family are HIV-infected [39]. Using models to approximate the effect of maternal HIV on young children in LMIC settings, it has been estimated that, in HIV-affected families, school completion falls from 61 to 57%, and children have a 10% higher incidence of anxiety or depression than HIV-unexposed children [99–102].

### HIV-Specific Pathways

Children born to HIV-infected mothers may be exposed to both HIV and ART antenatally, perinatally and/or postnatally. It is likely that in addition to the universal pathways discussed above, there are separate HIV-related mechanisms that impact on the child’s growth and development including (i) directly through exposure to HIV virions, (ii) through effects of maternal immune activation/inflammation on the in utero environment, (iii) by promoting immune activation/inflammation in the fetus and/or child and (iv) via ART toxicity throughout the period of in utero development and breastfeeding.

#### Direct Exposure to HIV Virions and Viral Proteins

HIV is a neurotropic virus, which can cause encephalopathy in HIV-infected infants, leading to microcephaly and neurodevelopmental impairment [21]. Neuropathology occurs directly, following exposure to HIV proteins that exhibit neurotoxicity, and indirectly via microglial activation and neuroinflammation. It is conceivable that antenatal exposure to HIV may affect brain development even without infection. Studies have detected HIV-specific immune responses in HEU children, suggesting that sufficient HIV antigen exposure occurs antenatally or perinatally to prime immune responses [103]. Direct exposure of the fetal brain to HIV virions and proteins may therefore be hypothesised to cause HIV-mediated neurotoxicity and impact brain growth and development*.* It is possible that in utero exposure to HIV virions may also modulate immune responses, resulting in adverse outcomes, similar to other chronic maternal infections including malaria, as discussed below [103].

#### Maternal Immune Activation/Inflammation

A healthy, regulated immune system is important for neurodevelopment [104]; therefore, HIV-related maternal immune activation and chronic systemic inflammation may impact fetal development. Advanced maternal HIV disease antenatally, which is driven by immune activation, has been consistently associated with HEU child morbidity [86]. Le Roux and colleagues recently found that the duration and severity of HIV viraemia in mothers during the antenatal period was closely related to developmental outcomes in their HEU infants, but immune activation was not assessed in this study [105]. Dysregulated immune mechanisms have been linked to various neurological disorders, and epidemiological associations exist between infections in pregnancy, maternal immune activation and schizophrenia, autism and epilepsy in offspring [106, 107], suggesting biological plausibility for this hypothesis in the setting of HIV. Further studies of HIV-positive mothers and HEU offspring are required in order to evaluate associations between inflammation in pregnancy and child development outcomes in these critical early years.

#### Immune Activation/Inflammation in the HIV-Exposed Uninfected Child

Immune activation and inflammation have been demonstrated in HIV-exposed fetuses and infants [108] which may trigger cytokine release, directly impacting cell migration and axonal growth and overall brain development [109–111, 112••]. Similarly, growth hormone axis disruption of Zimbabwean HEU children has been associated with systemic immune activation and the level of CMV replication [113]; inflammation in these infants was predominantly driven by higher HIV viral loads in mothers transmitting CMV [46]. Taken together, there appears to be a complex relationship between maternal HIV viraemia, early-life CMV acquisition, infant immune activation and the growth hormone axis, which may plausibly contribute to poor linear and brain growth in HEU children, and subsequent impaired neurodevelopment. Immune activation and inflammation may also drive neurodevelopmental impairment directly by impacting cell migration and axonal growth via proinflammatory cytokines [112••].

#### ART Toxicity

Antiretrovirals cross the placental barrier with varying concentrations, and their potential effects on offspring growth and development continue to be investigated [114]. It is challenging to disentangle the effects of HIV and ART, particularly because treatment improves maternal health, which may offset any negative impact. Although ART is clearly essential both for maternal health and to reduce vertical transmission of HIV, exposure has been associated with adverse outcomes in some studies [25], including prematurity, poor growth, metabolic disturbance [115–117] and mitochondrial abnormalities [118–120]. Other studies have found no serious adverse effects [121, 122]. The Pediatric HIV/AIDS Cohort study (PHACS) has established the Surveillance Monitoring for ART Toxicities in HEU children (SMARTT) study to monitor for ART toxicities across a range of metabolic, growth, cardiac and neurological outcomes [123, 124]. Results indicate little adverse effect of maternal ART on child outcomes [35]; however, atazanavir has been found to potentially impact language acquisition [25, 35, 36, 125], and tenofovir has been associated with an adverse impact on bone mineral content [126]. Recently, concerns have been raised over neural tube defects following dolutegravir exposure at conception [127•]. Children may additionally be exposed to ART via breast milk or directly as prophylaxis. Transient haematological alterations, including anaemia, have been associated with zidovudine exposure [128]. Further pharmacovigilance is needed to document the long-term safety of individual antiretroviral drugs and treatment combinations [108].

### Mechanistic Pathways Mediating Impaired Growth and Development

Universal and HIV-specific risk factors may drive poor growth and neurodevelopment through a common set of mechanistic pathways, as outlined in Fig. 2, including co-infections, inflammation, enteropathy, anaemia, nutrient deficiencies, epigenetic modifications and toxic stress, ultimately impacting brain development. However, we lack data on many of these pathogenic processes, meaning studies are needed to further our understanding of the pathways within this conceptual framework. Here, we provide some insights that support these mechanisms as potential mediators of the risk factors discussed above.

Direct exposure to opportunistic infections may cause neurotoxicity and impair growth. Congenital CMV infection in particular has been shown to have a profound impact on neurodevelopment, and the outcomes and mechanisms have been reviewed comprehensively elsewhere [129]. CMV acquisition in early life is very common in sub-Saharan Africa. The impact of CMV on growth and development may be even greater in HEU children and has recently been reviewed [45•]. Garcia-Knight and colleagues found that CMV viral load in early infancy was negatively associated with weight-for-age and head circumference-for-age *Z* scores in both HIV-exposed and HIV-unexposed children in rural Kenya [130], and Gompels and colleagues found that CMV was associated with growth and early child development in Zambia, particularly amongst those exposed to HIV [47].

Environmental enteric dysfunction (EED) is an almost ubiquitous subclinical disorder of the small intestine in LMIC. EED is characterised by villous atrophy, impaired gut barrier function, intestinal inflammation and microbial translocation, leading to systemic inflammation [131]. We have previously hypothesised that EED may be more severe amongst HEU compared to HIV-unexposed children [132], although a study of Zimbabwean infants at 6 weeks and 6 months of age showed similar levels of intestinal fatty acid binding protein (I-FABP), a marker of enterocyte damage, in HEU and HIV-unexposed infants. However, CRP was consistently higher in HEU compared to HIV-unexposed infants, highlighting that systemic inflammation is greater in the setting of HIV exposure, although the drivers of inflammation remain poorly defined [133]. Proinflammatory cytokines appear to be upregulated in HEU compared to HIV-unexposed children even at birth, and certain brain maturation processes such as cell migration and axonal growth may be particularly vulnerable to this inflammatory milieu [111].

A common co-morbidity with stunting is iron deficiency anaemia (IDA). IDA is a major cause of neurodevelopmental impairment [43] and has been identified as one of the leading causes of years lived with disability in children [134]. HIV-exposed children have a higher frequency of anaemia than HIV-unexposed children [135, 136], plausibly driven by exposure to the virus itself and/or exposure to ART [137]. HIV infection is associated with other micronutrient deficiencies [52], and similar effects may be seen in HEU children, although data are currently lacking.

Early-life programming may be influenced by the in utero environment, causing DNA methylation and gene expression modifications [138]. These epigenetic modifications are now understood to have potential biological impact across the lifespan, leading to the theory of the developmental origins of health and disease and may have intergenerational effects [139]. HIV infection has been shown to lead to epigenome-wide differential DNA methylation in infected individuals [140]. Additionally, some studies have indicated that maternal HIV infection is associated with epigenetic modifications in neonates, and HIV and ART-exposed children have been found to have reduced DNA methylation in peripheral blood repetitive elements which may have long-term implications [141]. Further work is needed to understand this area; however, research into HIV-associated neurocognitive disorders (HAND) in adults indicates a role for genetic and epigenetic profiles in predicting vulnerability to the neurological effects of the virus and ART side effects [142].

Children living in adverse environments are at risk of chronic stress and persistent activation of their physiological stress response—a process known as toxic stress [143]. This process may act via the hypothalamic–pituitary–adrenal (HPA) axis and immune responses to disrupt healthy brain circuit development, particularly in the prefrontal cortex [144]. Through this impact on the neuroendocrine–immune (NEI) network, early experiences can fundamentally shape the developing brain architecture [143]. Early mutual interactions and experiences between children and key adults also shape the developing brain architecture and affect the NEI network [143]. Maternal physical and psychological illness coupled with a lack of social support may impact on a mother’s ability to provide a safe and secure physical and emotional environment for her infant [145]. Similarly, growing up in an environment with inadequate stimulation or few early learning opportunities is associated with reduced cognitive development [146]. Children growing up in families affected by HIV may face multiple adversities that affect the parent–child relationship including parental illness and death, mental illness, and stress from emotional, financial and social pressures [147].

Ultimately, the risk factors and mechanisms identified here may modulate brain growth and network development. In addition, there may be other risk factors and mechanisms, including those as yet unknown, influencing these children. The in utero period and early postnatal life is a time of substantial brain growth, when extensive neural network development and maturation take place [7]. The developing brain during this time is particularly sensitive to environmental influences [148]. Animal models as well as human studies suggest that infection-induced maternal immune activation impacts developing neural circuits [106]. Studies have indicated that maternal immune activation and induction of proinflammatory cytokines affecting the microbiota–gut–brain axis or eliciting the stress response through the HPA axis result in atypical brain development of the fetus [107, 149, 150]. Neuroimaging study findings of HEU-associated white matter microstructural changes early in life [110, 151] suggest abnormal brain development as a potential mechanism for impaired child outcomes.

## Improving HEU Child Growth and Developmental Outcomes

Healthy birth, growth and development form the foundations for later school performance, employment opportunities and long-term human capital and health. First and foremost, the continued global focus on reducing the burden of antenatal HIV infection is the key to eliminating HIV exposure in children. However, given the expanding population of HEU children, efforts that focus on prevention and intervention strategies to reduce the burden of poor HEU child outcomes are also needed [108].

### Prevention

In order to develop successful prevention strategies, further research is needed to understand the effects of HIV exposure on child outcomes. Improved ART coverage in recent years means that family and home environments may be different to previous decades; infant feeding advice has shifted over time and the impact of breastfeeding patterns in this population is still being unravelled; children are now more commonly exposed to ART in utero and postnatally, and more women are conceiving on ART. Studies examining the relative contributions of HIV-related pathways to growth and developmental impairment in the context of universal risk factors in the era of ART are needed to inform and focus these strategies [93]. In order to detect early adverse outcomes and intervene, pharmacovigilance systems are needed to assess effects of ART on the growing and developing child. This is particularly relevant with the introduction of new drugs and regimens, and new approaches to prevent HIV infection in pregnant and breastfeeding women using pre-exposure prophylaxis (PrEP). The concerns raised over dolutegravir safety at the time of conception provide lessons going forward [63]. Evaluating the relative safety of different ART regimens in pregnancy for HEU child growth, brain health and development is critical.

Some studies suggest there may be a high-risk subgroup within the HEU population vulnerable to adverse outcomes. Studies have found interactions between HIV exposure and preterm birth leading to poorer growth [19•] and worse cognitive and motor outcomes [29•], and also poorer outcomes in HEU children with CMV coinfection, compared to those without [47]. Further evidence is needed to define these high-risk subgroups, who may benefit from targeted prevention strategies, particularly where resources are limited. Initiating prevention before conception to optimise the health of mothers and their families will likely be most effective.

### Intervention

Alongside the development of prevention policies, intervention strategies are needed to identify and support HEU children at risk of growth and developmental impairment to improve long-term outcomes. Evidence from institutionalised children suggests interventions need to be initiated early (before the age of 5 years) to reverse impairment during critical periods of growth and development; however, the optimal window of opportunity for HEU children remains unclear [43].

Targeting the universal risk factors that impact growth and development is a logical strategy, through improved breastfeeding, adequate nutrition, support for maternal physical and psychosocial health, reducing exposure to toxins, prevention and early treatment of childhood infections and reducing poverty. Integrating this with improved maternal ART adherence throughout the period of pregnancy and breastfeeding to ensure HIV viral suppression would also likely contribute to improved outcomes amongst HEU children. This may be delivered as integrated care through multisectoral platforms targeting health, nutrition and education, involving both the caregiver and the child to break the intergenerational cycle, using approaches such as the UNICEF Nurturing Care Framework.

Concurrently, research informing additional targeted interventions to address the HIV-related pathways by assessing scalable packages of care for early child growth and development are needed. Recently, the SHINE trial in rural Zimbabwe demonstrated that the combination of improved infant and young child feeding and improved water, sanitation and hygiene interventions resulted in better outcomes in both motor and language development amongst HEU children [152]. Further work is required to examine the most effective, scalable intervention strategies moving forward.

## Conclusions

There are currently 14.8 million HEU children worldwide and this population continues to expand. Increasing evidence suggests these children, particularly in LMIC settings, have a disparity in growth and development compared to HIV-unexposed children. Our conceptual framework highlights potential pathways linking HIV and/or ART exposure and adverse outcomes. We propose HEU children may be affected by accentuating existing universal risk factor pathways as well as through HIV-specific pathways, via final common pathogenic mechanisms*.* Overall, a multifactorial causal pathway is most likely to shape the growth and neurodevelopment of the HEU child. More research is critically needed in the era of universal ART, to understand these pathways further and inform prevention and intervention strategies.
